# Evolutionary polynomial regression improved by regularization methods

**DOI:** 10.1371/journal.pone.0282029

**Published:** 2023-02-17

**Authors:** Yao Li, Mo Li, Lei Zhang

**Affiliations:** 1 School of Geosciences and Surveying Engineering, China University of Mining and Technology-Beijing, Beijing, P.R. China; 2 State Key Laboratory Coal Resources and Safe Mining, China University of Mining and Technology-Beijing, Beijing, P.R. China; 3 School of Civil Engineering and Mechanics, Yanshan University, Qinhuangdao, Hebei, P.R. China; Semnan University, ISLAMIC REPUBLIC OF IRAN

## Abstract

Evolutionary polynomial regression (EPR) is a data mining tool that has been widely used in solving various geotechnical engineering problems. The fitness function is the core of EPR. However, overfitting may still occur in EPR, and this issue may cause the testing dataset not to perform effectively. Improvement of the EPR fitness function through L1 and L2 regularization methods is critical to avoid overfitting and enhance good generalization. First, the appropriate values of the regularization parameter *λ* of the L1 regularization method (L1RM) and L2 regularization method (L2RM) are determined by comparing the test data sets. Then, the EPR with a classical fitness function is compared with that of L1 or L2 regularization methods to evaluate their abilities in developing regression and producing accurate predictions. The results show that the fitness function combined with the regularization method could improve the EPR. However, L1RM performs better in prediction than L2RM. Improvement of EPR using L1RM could solve problems associated with construction constitutive models or could be used for prediction in geotechnical engineering.

## 1. Introduction

Evolutionary polynomial regression (EPR) is a numerical regression method that was first developed in 2006 [1]. Recently, EPR has been successful in modeling nonlinear complex problems. As presented in a study [2], the genetic algorithm (GA) [3] is used to construct the general functional structure from elementary functions in EPR. Additionally, the parameter *a*_*j*_ is estimated by the least squares method (LSM). In 2009, Javadi and Rezania [4, 5] were the first to use EPR to solve problems associated with the geomaterial constitutive model. Ahangar-Asr *et al*. [6] presented a new approach using EPR in 2011 for predicting permeability, maximum dry density, and optimum moisture content as functions of some physical soil properties. Faramarzi *et al*. [7] developed material models for soils based on EPR, in which volumetric behavior was taken into account. Javadi *et al*. [8] proposed a new approach to implementing the finite element method using EPR for modeling soil behavior under cyclic loading. Cuisinier *et al*. [9] analyzed the impact of high-pH fluid on the mechanical behavior of backfill material for deep galleries via the EPR model.

The EPR model is used to construct the geomaterial constitutive models. It is also used as a regression model to find the best model to fit the observed data and solve geotechnical problems. Ahangar-Asr *et al*. [10] analyzed the stability of soil and rock slope with EPR, and the results showed that the proposed approach was robust in modeling the slope behavior and in providing a unified approach for the analysis of the slope stability problems. Doglioni *et al*. [11] introduced the EPRMODA to model the relationship between the expected displacements, the past measured values of displacement, and past cumulative rainfall values. Wu *et al*. [12] combined the EPR and real coded genetic algorithm to correlate the compression index with the physical properties of the clay. Yin *et al*. [13] proposed a new approach for evaluating the compressibility of remodeled clay using the EPR and optimization methods. Rezania *et al*. [14] introduced a new approach using EPR for determining the liquefaction potential of sands. Rezania *et al*. [15] developed another model based on EPR for evaluating the potential of liquefaction and lateral spreading. Balf *et al*. used EPR in one-dimensional water quality models to improve the simulation of solute concentration in natural rivers [16]. EPR is also effective for geotechnics applications for predicting soil saturated water content [17], evaluating pile-bearing capacity [18, 19], and conducting the description of soil behaviors [20]. Thus, EPR has proved to be more robust in solving geotechnical problems than soft computing methods, such as artificial neural networks (ANNs) [21, 22], or genetic programming (GP) [23].

However, in most current EPR models, fitness functions suffer from training data errors, such as the sum of squared errors (SSE) or the coefficient of determination. As a result, the proposed models are usually overfitting and weak in terms of generalization ability [24]. Thus, the EPR technique must be improved to avoid overfitting and to achieve good generalization ability. Nikolaev and Iba [25] presented a regularization approach for inductive genetic programming, tuned for learning polynomials. Montana *et al*. [26] presented a regularization method for controlling program complexity in linear genetic programming, tuned for transcendental elementary functions. Their goal was to improve the performance of evolutionary methods when solving symbolic regression tasks. Jin *et al*. [24] proposed a novel EPR procedure using a newly enhanced differential evolution (DE) algorithm. Maracco *et al*. [27] presented a robust and well-conditioned EPR technique to remove potential outliers, leverage points, and any biased data set. Roozbeh, Babaie–Kafaki, and Aminifard [28, 29] used different techniques to improve the modern regression method.

This study proposes an improved EPR method to enhance the fitness function through the regularization method. First, we determine the appropriate value of the regularization parameter (*λ*) of the L1 regularization method (L1RM) and L2 regularization method (L2RM) by comparing the test data. Then, we compare EPR with the classical fitness function using a combination of L1RM and L2RM to test their regression and prediction performances. Finally, we compare and analyze the EPR method and enhanced fitness function to determine their level of improvement.

## 2. Classical EPR and regularization methods

### 2.1 Overview of classical EPR methods

EPR is a numerical regression method based on evolutionary theory aimed at identifying the best polynomial expressions between input and output data. The classical form of the EPR is presented in (1) [1]:

$$Y_{N\times 1}(\theta ,Z)=\left[\begin{array}{cc} I_{N\times 1} & Z_{N\times m}^{j} \end{array}\right]\times {\left[\begin{array}{cccc} a_{0} & a_{1} & \cdots & a_{m} \end{array}\right]}^{T}=Z_{N\times d}\times \theta_{d\times 1}^{T}$$ (1)

where **Y**_*N*×1_(**θ**,**Z**) is the least squares estimation of the vector *N* target values; **θ**_*d*×1_ is the vector of *d = m + 1* parameters *a*_*j*_; *j = 1*:*m*, *m* is the number of terms, and *a*_0_; **Z**_*N*×*d*_ is a matrix formed by **I**, a unitary column vector for bias *a*_0_, and m vectors of variables **Z**^*j*^. A fixed *j* is a product of the independent predictor vectors of variables/inputs. **X** = [**X**_1_ **X**_2_ ⋯ **X**_*k*_], where k is the dimension of the independent variables.

The matrix of inputs **X** is given as follows:

$$X=\left[\begin{array}{ccccc} x_{11} & x_{12} & x_{13} & \ldots & x_{1k}\\ x_{21} & x_{22} & x_{23} & \ldots & x_{2k}\\ x_{31} & x_{32} & x_{33} & \ldots & x_{3k}\\ \ldots & \ldots & \ldots & \ldots & \ldots \\ x_{N1} & x_{N2} & x_{N3} & \ldots & x_{Nk} \end{array}\right]=\left[\begin{array}{ccccc} X_{1} & X_{2} & X_{3} & \cdots & X_{k} \end{array}\right]$$ (2)

where the k-th column **X** denotes the candidate variables for the j-th term in (1). Therefore, the j-th term in (1) is written as:

$$Z_{N\times 1}^{j}=\left[{\left(X_{1}\right)}^{ES(j,1)}\cdot {\left(X_{2}\right)}^{ES(j,2)}\cdot {\left(X_{3}\right)}^{ES(j,3)}\cdot \cdots \cdot {\left(X_{k}\right)}^{ES(j,k)}\right],\;\forall j=1\ldots m$$ (3)

where **Z**^*j*^ is the j-th column vector whose elements are products of candidate-independent inputs, and **ES** is a matrix of exponents. The (1) is expanded as follows:

$$\left(\begin{array}{c} Y_{1}\\ Y_{2}\\ \vdots \\ Y_{N} \end{array}\right)=\left[\begin{array}{ccccc} 1 & Z_{11} & Z_{12} & \cdots & Z_{1m}\\ 1 & Z_{21} & Z_{22} & \cdots & Z_{2m}\\ \vdots & \vdots & \vdots & \ddots & \vdots \\ 1 & Z_{N1} & Z_{N2} & \cdots & Z_{Nm} \end{array}\right]•\left(\begin{array}{c} a_{0}\\ a_{1}\\ a_{2}\\ \vdots \\ a_{m} \end{array}\right)$$ (4)

The key idea of the EPR is to start from (1) and use GA to search for the best form of the function first, i.e., a combination of vectors of independent variables (inputs), **Z**_*N*×*d*_. The next step is to determine the adjustable parameters **θ** (vector of *a*_*j*_) for each combination of inputs using the least squares regression model. When GA is searching, Giustolisi and Savic [1] propose the penalization of complexity (PCS) fitness function for the trade-off between the quality of the SSE fit and the model complexity (number of input combinations), as shown below:

$$PCS=\frac{SSE}{N\cdot {\left(Nd-px+1\right)}^{a}}$$ (5)

with

$$SSE={\sum_{i=1}^{N}\left({\overset{\wedge }{y}}_{i}(\theta ,Z)-y_{i}\right)}^{2}$$ (6)

where *Nd* = *k* • *m* is the maximum number of inputs that can be considered, *px* is the actual number of inputs selected by the GA, and *a* is an adjustable exponent that controls the degree of pressure to control complexity. Moreover, ${\overset{\wedge }{y}}_{i}\left(\theta ,Z\right)$ is the prediction value computed by the polynomial expression and obtained by EPR; *y*_*i*_ is the target value of the training data set. The complexity of the regression model is reduced to control the total number of inputs in (5). However, the risk associated with model overfitting must still be studied.

### 2.2 Regularization method

The regularization/penalty functions are common practice in machine learning and are generally applied in GA [30]. L1 regularization and L2 regularization are some of the regularization techniques mostly used to address over-fitting and feature selection and create a less complex (parsimonious) model when having numerous features in a dataset.

L1 regularization, also known as the L1 norm or Lasso regression, adds the absolute value of the magnitude of the coefficient as a penalty term to the loss function [31] (like the fitness function in GA) as revealed below:

$$L=\frac{1}{N}{\sum_{i=1}^{N}\left({\overset{\wedge }{y}}_{i}-y_{i}\right)}^{2}+\lambda \sum_{i=1}^{N}\left|\theta_{i}\right|$$ (7)

where ${\overset{\wedge }{y}}_{i}$ is the predicted value, *y*_*i*_ is the actual value, *λ* is the regularization parameter, and *θ*_*i*_ is the weight/coefficient of the model term.

L2 regularization, also known as the L2 norm or Ridge regression, combats overfitting by forcing weights/coefficient values to be small. However, this does not make them exactly 0 because it is vital to add the sum of squares of all of the feature weights as a penalty term to the loss function:

$$L=\frac{1}{N}{\sum_{i=1}^{N}\left({\overset{\wedge }{y}}_{i}-y_{i}\right)}^{2}+\lambda {\sum_{i=1}^{N}\left(\theta_{i}\right)}^{2}$$ (8)

The penalty term is the key difference between the L1 and L2 two regularization methods. L1 regularization penalizes the sum of absolute values of the coefficients, whereas L2 regularization penalizes the sum of squares of the coefficients. As a result, L1 regularization shrinks the less important feature’s coefficient to 0 and removes some features altogether. In other words, L1 regularization works effectively for feature selection in cases for many features. However, L2 regularization does not perform feature selection since coefficients are only reduced to values near 0. Nevertheless, the robustness and generalization ability of the regression model is improved with the L1 or L2 regularization.

The value of the regularization parameter *λ* is vital to the regularization method. As shown in (7) and (8), if *λ* is 0, the loss function is the mean squared error (MSE). If the *λ* has a large value, the coefficient will be 0, and the regression model will be underfitting. The value of the *λ* is determined first through comparison and discussion.

## 3. EPR improved by regularization methods

### 3.1. New fitness function combined with L1RM and L2RM

The fitness function is the core of EPR because it helps GA find the best exponent matrix that determines the structure of the polynomial regression model. When the structure of the polynomial regression model is determined through the LSM, the regression model is obtained uniquely. Therefore, the performance of an EPR depends on the fitness function, as shown in (5). Thus, the classical fitness function (PCS) of EPR uses ‘(*Nd* − *px* + 1)’ to penalize the complexity of the regression model by reducing the actual number of inputs (the actual number of polynomial terms). Differently, fitness functions of improved EPR combined with L1RM or L2RM penalize the complexity of the regression model by reducing the values of the coefficients to address over-fitting (as mentioned in Subsection 2.2). The two new fitness functions combined with L1RM and L2RM are expressed as follows:

$$L1RM=\frac{1}{N}\cdot SSE+\lambda \sum_{i=1}^{N}\left|\theta_{i}\right|$$ (9)

$$L\text{2}RM=\frac{1}{N}\cdot SSE+\lambda {\sum_{i=1}^{N}\left(\theta_{i}\right)}^{\text{2}}$$ (10)

where *N* is the number of data points and SSE is the sum of squared errors calculated by (6). Then, *λ* is the regularization parameter and *θ*_*i*_ is the coefficient of the regression model.

When EPR is working, a random exponent matrix is first generated. The experimental EPR model is obtained through LSM. Then, the fitness function is calculated to determine whether the EPR model is optimal. In this process, the goal is to make the error of the test model as small as possible and to accurately predict new samples. However, this can still cause overfitting problems. A generalization method such as L1RM can solve this problem; L1RM controls overfitting through a sparse coefficient vector, and it adds the sum of the absolute values of the coefficient vector to the fitness function.

### 3.2 Discussion of regularization parameters

**Fig 1:**
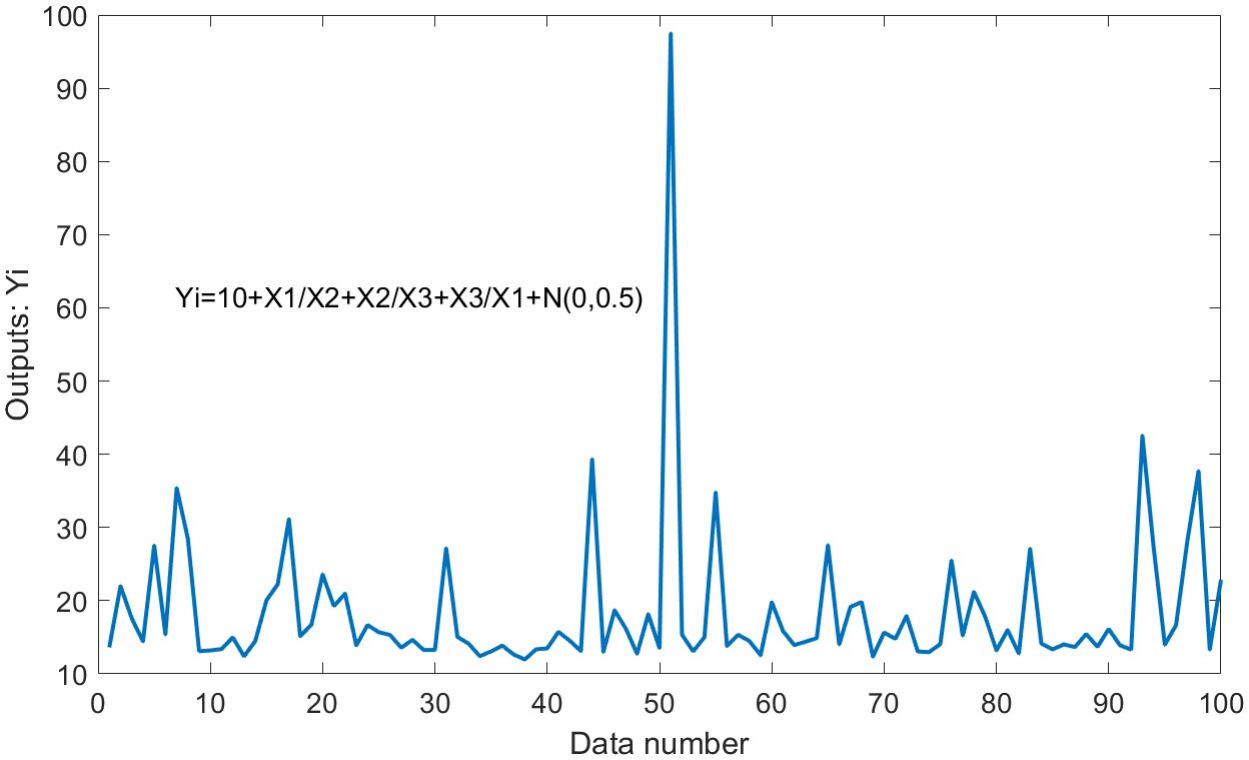
Outputs Y with errors in dataset 1.

**Fig 2:**
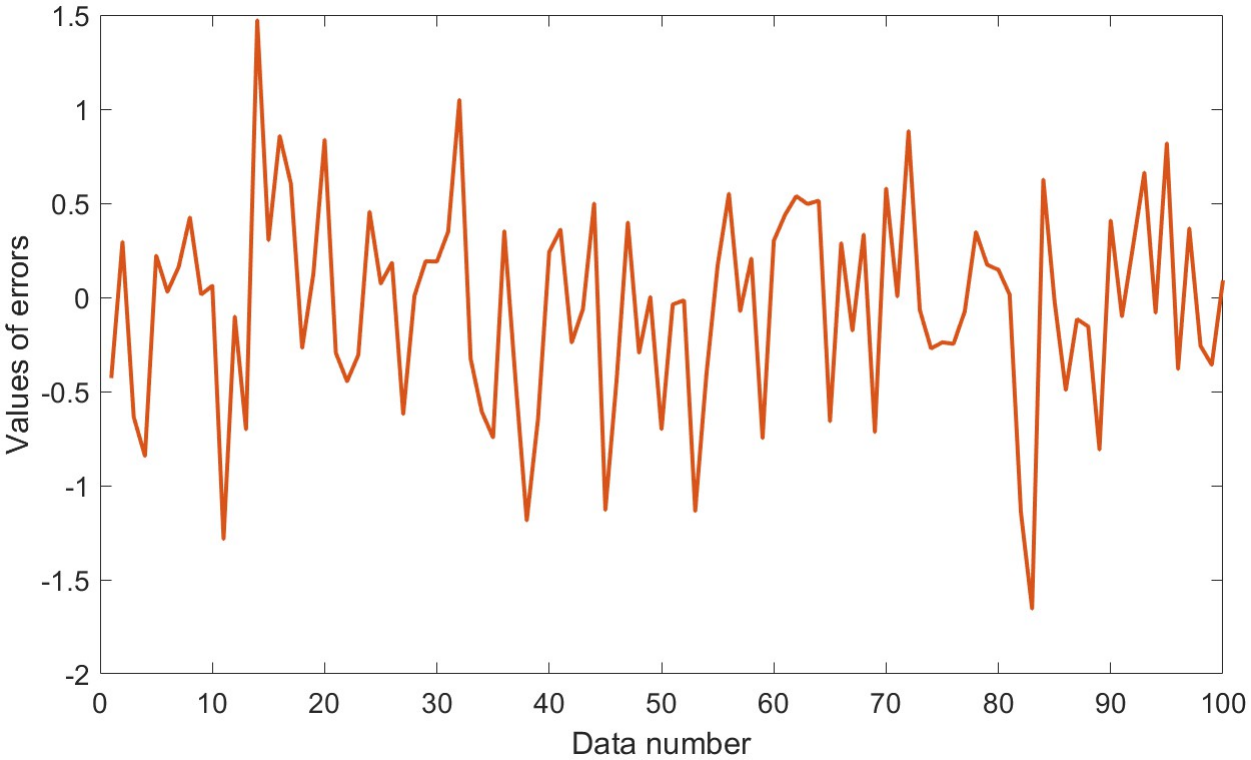
Values of errors in dataset 1.

The regularization parameter *λ* is vital to the regularization method; this is discussed first, and a 100-testing dataset (dataset 1) was generated by (11) [1]:

$$Y=10+1\cdot X_{1}/X_{2}+1\cdot X_{2}/X_{3}+1\cdot X_{3}/X_{1}+N(0,\sigma )$$ (11)

where **Y** plays the role of outputs; **X**_1_, **X**_2_, and **X**_3_ are three random variables (uniformly distributed random numbers in the range [0,1]), which play the role of inputs. Moreover, *N*(0, *σ*) is normally distributed random numbers having zero mean and standard deviation *σ*, which play the role of the error term. Thus, the standard deviation *σ* is equal to 0.5. Some statistical values for **Y** and **Z**_*i*_ (**Z**_1_ = **X**_1_/**X**_2_, **Z**_2_ = **X**_2_/**X**_3_, **Z**_3_ = **X**_3_/**X**_1_) are shown in Table 1. Outputs **Y** with errors and the values of errors are shown in Figs 1 and 2.

**Table 1:** Statistics of output Y and terms Z_i_ (dataset 1).

	Y	Z1	Z2	Z3
Maximum	97.530	19.783	86.821	30.689
Minimum	13.012	0.028	0.044	0.017
Mean	18.389	2.032	3.220	3.137
Variance	104.210	10.492	88.134	35.538

The value of the exponent was constrained to integers in the range [-2, 2]; the number of terms was constrained to [2, 8] for the generalization parameter. For GA, the population size was set to 80; the crossover probability rate was set to 0.4, and the mutation probability rate was set to 0.1. The coefficient of determination (R^2^) was selected to evaluate the performance of the obtained model. Higher R^2^ values are closer to 1, showing that the performance is better.

**Fig 3:**
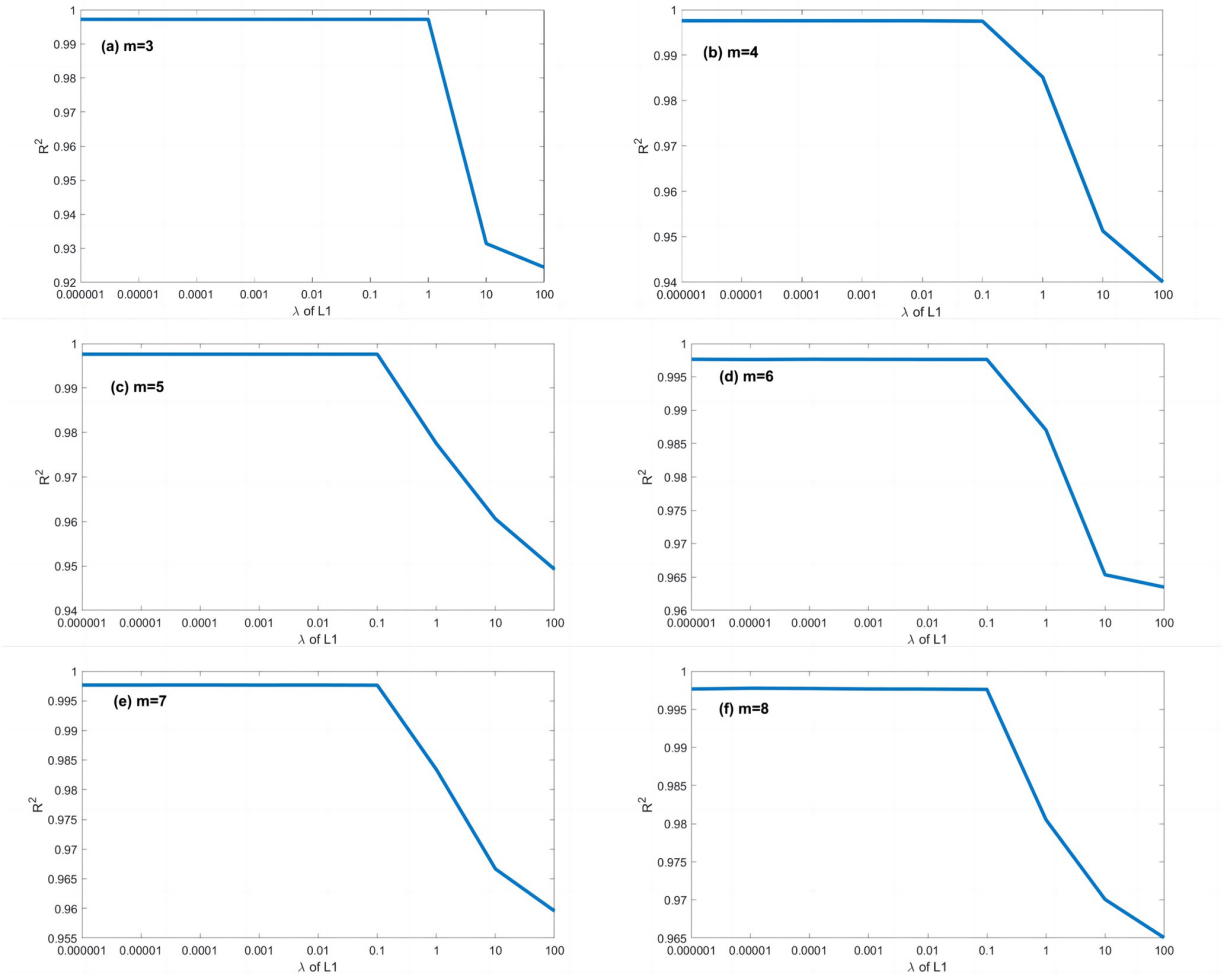
Comparison of EPR results (R^2^) for different values of λ to L1RM (dataset 1). (a) m = 3, (b) m = 4, (c) m = 5, (d) m = 6, (e) m = 7 and (f) m = 8.

**Fig 4:**
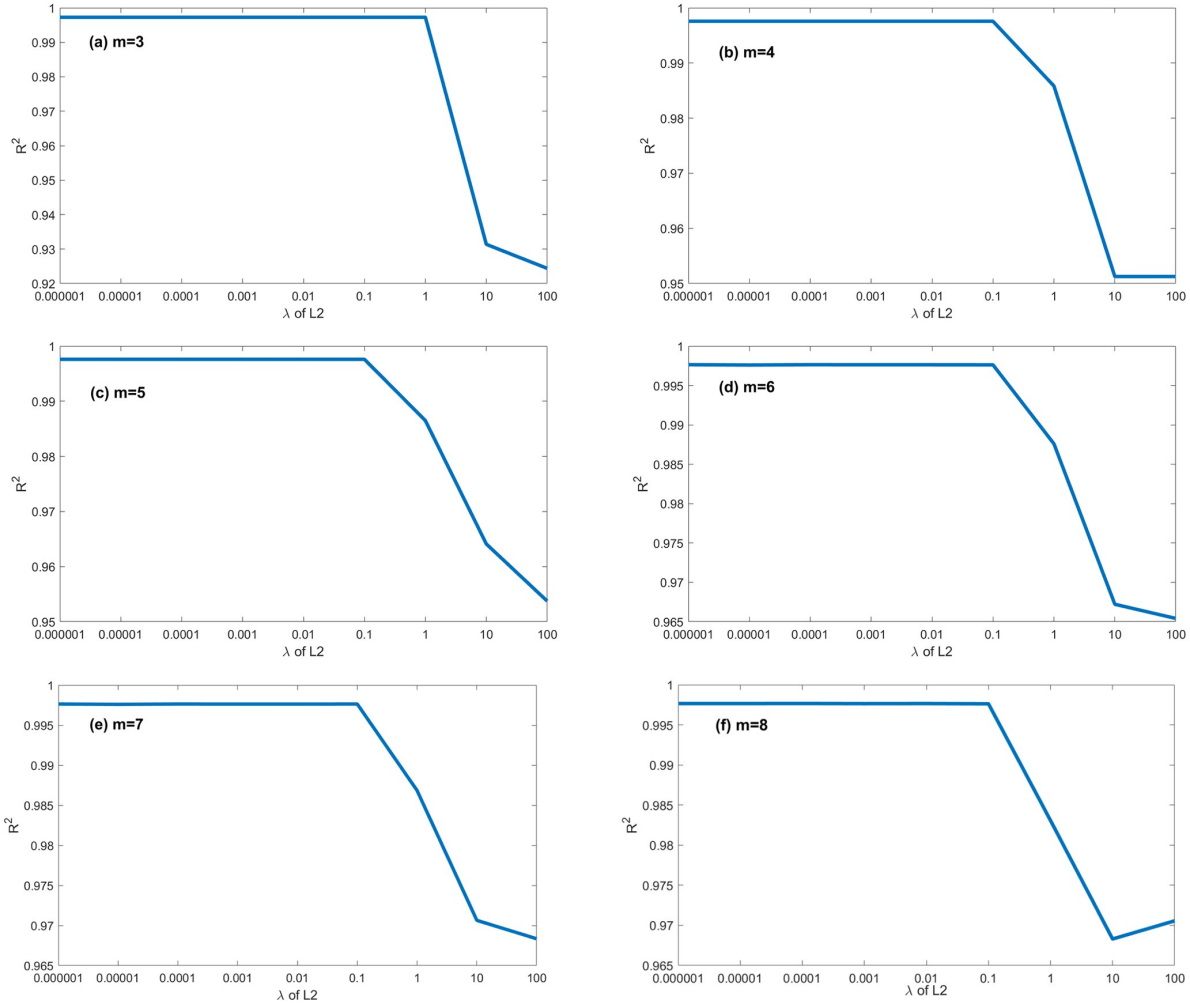
Comparison of EPR results (R^2^) for different values of λ to L2RM (dataset 1). (a) m = 3, (b) m = 4, (c) m = 5, (d) m = 6, (e) m = 7 and (f) m = 8.

A series of calculations using different values of *λ* (i.e., *λ* = 0.000001, 0.00001, 0.0001, 0.001, 0.01, 0.1, 1, 10, 100) is conducted to evaluate how *λ* affects the EPR when combined with L1RM and L2RM. The values of *λ* were determined according to the mean value of output (Y), i.e., the max value was 10 times the order of magnitude of Y, and the min value was 10^−7^ times the order of magnitude of Y. Figs 3 and 4 show that the results (R^2^) of different *λ* of L1RM and L2RM ranges from 3 to 8 when some terms (represented by “m” in the figures).

As shown in Figs 3 and 4, R^2^ increases. In contrast, the *λ* of L1RM and L2RM decreases from 100 to 0.000001. In most cases, when *λ* decreases to 0.1, R^2^ will reach the top and remain at the stable maximum value. The appropriate value of *λ* is that the R^2^ premises are large (close to 1), revealing that the trade-off between performance and generalization is obtained. Moreover, the performance of EPR does not improve significantly with an increase in the number of terms because of the simple data structure and small errors. Next, a more complex dataset was created to simulate the elastic constitutive model of geotechnical engineering and to test the EPR performance with the regularization method.

### 3.3. Performance of EPR with L1RM and L2RM

#### 3.3.1. Determination of the appropriate value of *λ*

**Fig 5:**
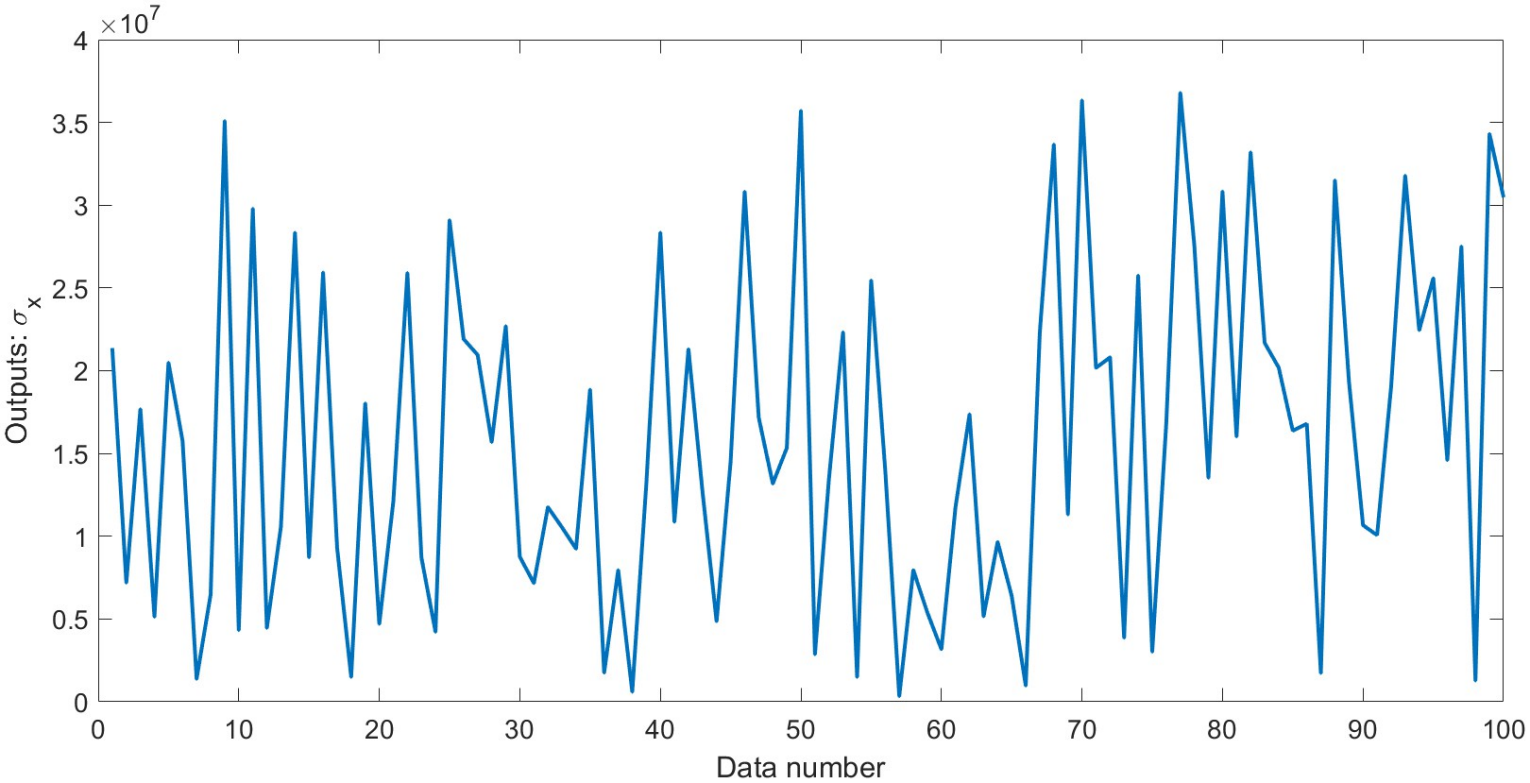
Outputs *σ*_*x*_ with errors in dataset 2.

**Fig 6:**
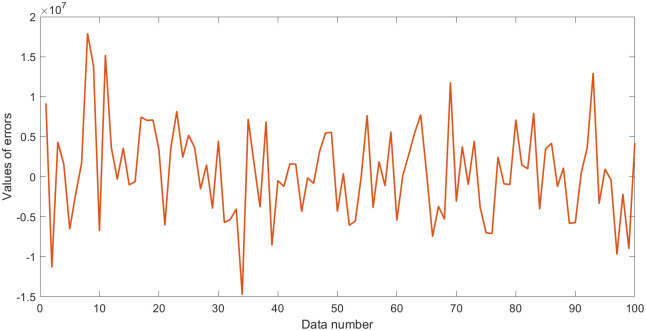
Values of errors in dataset 2.

In this section, a 100-testing dataset (dataset 2) was generated by the elastic constitutive Eq (12) of geotechnical engineering. The values of the parameters and variables in (12) were randomly generated in the specified intervals, similar to the engineering values, to test the performance of EPR with L1RM and L2RM, as shown in Table 2. Thus, the appropriate value of *λ* to the elastic dataset must be determined because of the different orders of magnitude and the complexity. The value of *λ* ranges from 1 to 1*10^8^, intervals of ten times, according to the determination rules in Subsection 3.2. Other EPR parameters are the same as those in Subsection 3.2. Therefore, the coefficient of determination (R^2^) was selected to evaluate the performance of the obtained model. Larger errors were used as shown in the statistics in Figs 5 and 6.

$$\sigma_{x}=E\cdot \frac{\varepsilon_{x}+\mu \cdot \varepsilon_{y}}{1-\mu^{2}}+{\text{10}}^{\text{7}}\cdot N(0,0.5)$$ (12)

where *σ*_*x*_ is the stress in the x-axis direction; *E* is the elastic modulus; *ε*_*x*_ and *ε*_*y*_ is the strain in the x-axis direction and y-axis direction, respectively; *μ* is Poisson’s ratio; and *N*(0, *σ*) is normally distributed random numbers having zero mean and standard deviation *σ*, which plays the role of the error term.

**Table 2:** Statistics of the parameters and variables in dataset 2.

	*σ*_*x*_	*E*	*ε*_*x*_	*ε*_*y*_	*μ*
	(*10^7^ N/m^2^)	(*10^8^ N/m^2^)			
Maximum	3.679	3.988	0.0995	0.0097	0.396
Minimum	0.0329	2.004	0.00009	0.00018	0.200
Mean	1.978	3.002	0.043	0.0054	0.306
Variance	1.137*10^7^	3.580*10^7^	0.00077	0.000008	0.0034

**Fig 7:**
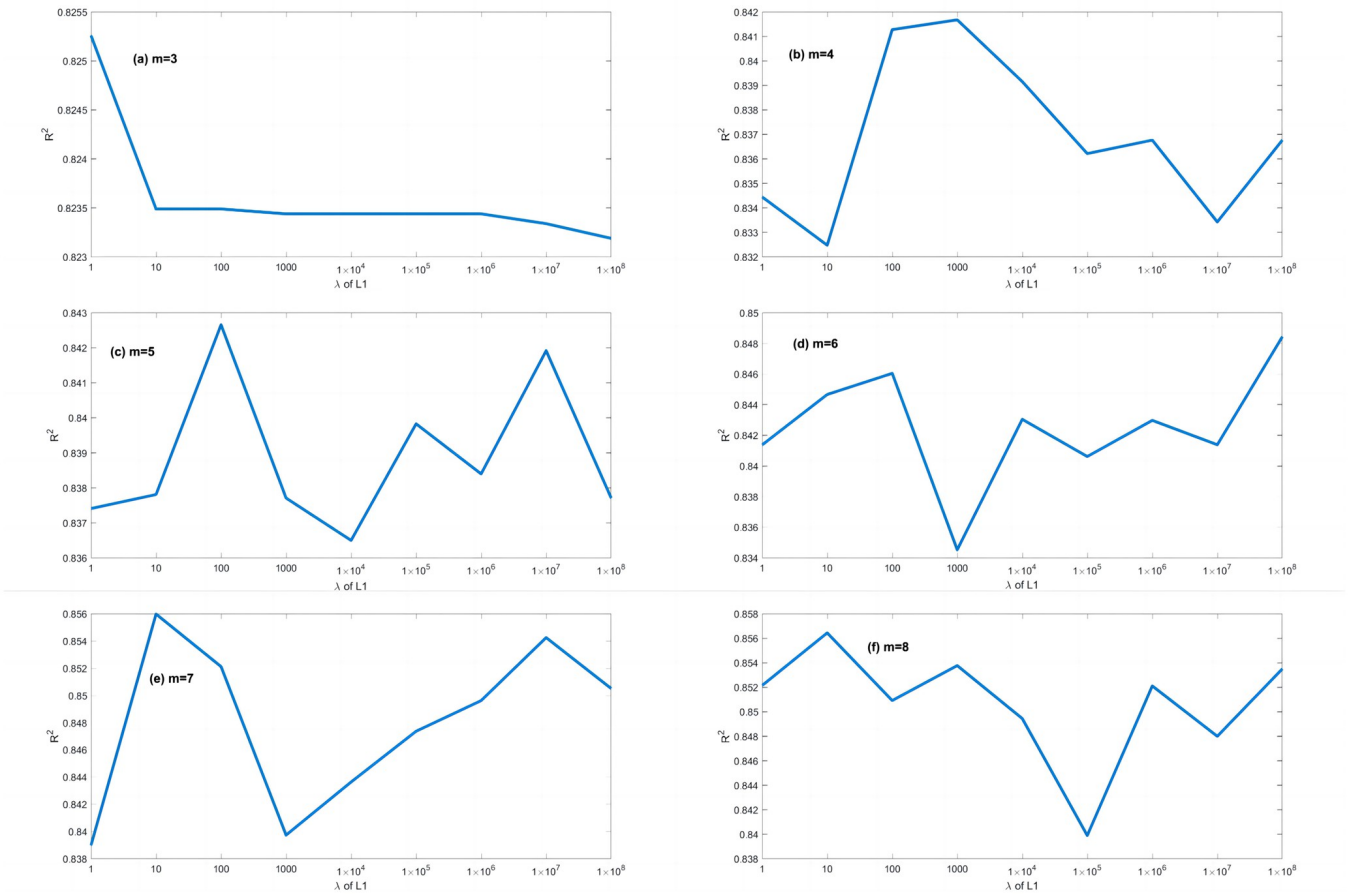
Comparison of EPR results (R^2^) in different values of λ to L1RM (dataset 2). (a) m = 3, (b) m = 4, (c) m = 5, (d) m = 6, (e) m = 7 and (f) m = 8.

**Fig 8:**
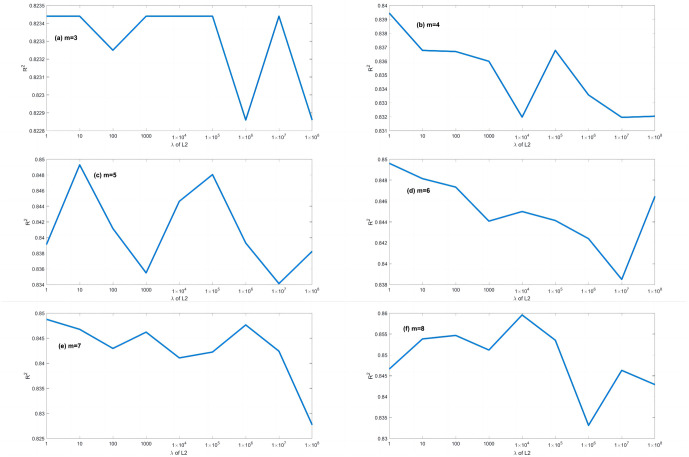
Comparison of EPR results (R^2^) in different values of λ to L2RM (dataset 2). (a) m = 3, (b) m = 4, (c) m = 5, (d) m = 6, (e) m = 7 and (f) m = 8.

Figs 7 and 8 compare the EPR results (R^2^) of different values in λ with L1RM and L2RM, respectively. Since the effect of the error was larger, the regression results (R^2^) were volatile. Moreover, the change of λ in L1RM and L2RM was irregular. The rules of finding the appropriate λ are still the same, i.e., the R^2^ premise is large (closer to 1); λ is also as large as possible. As shown in Fig 7, when λ is equal to 100, the R^2^ is always at a high level, revealing that the appropriate value of λ to L1RM is 100. As shown in Fig 8, when λ is equal to 10^5^, the R^2^ is always at a high level, and the appropriate value of λ to L2RM is 10^5^. When λ was equal to 0, the results (R^2^) of EPR (whose fitness function was only MSE, without regularization method) were listed and compared with that of the regularization method having an appropriate λ (Table 3). When m is less than 5, the results (R^2^) of EPR with a regularization method having an appropriate λ are very close to those whose λ is equal to 0 (Table 3); some are even better. When m is ≥5, the differences in results (R^2^) become slightly larger, but the largest relative difference is 2.5%, which is still small enough.

**Table 3:** Comparisons of R^2^ with different λ.

M (number of terms)	R^2^ of EPR without regularization (λ = 0)	R^2^ of EPR with L1RM having an appropriate λ	R^2^ of EPR with L2RM having an appropriate λ
3	0.82554	0.82349	0.82344
4	0.83563	0.84128	0.83677
5	0.85499	0.84266	0.84805
6	0.8602	0.84605	0.84414
7	0.86402	0.85212	0.84225
8	0.8631	0.85092	0.85353

#### 3.3.2 Performance of EPR with L1RM and L2RM

After the appropriate regularization parameters (λ) of the two regularization methods were determined, the performance of EPR with L1RM and L2RM was discussed and compared with classical EPR with the fitness function of PCS (5). Using (12), two different sizes of datasets were built, i.e., a 30-dataset (dataset 3) with 20 for regression and 10 for prediction. Thus, a 100-dataset (dataset 4) was used, in which 70 was for regression and 30 for prediction. All of the parameters and variables in the two datasets were randomly generated in the specified intervals, similar to the engineering values. Then, three indicators were used to evaluate the performance of the three EPR models obtained by three different fitness functions (the classical fitness function (PCS), the two improved fitness functions with L1RM and L2RM), i.e., coefficient of determination (R^2^), root mean square error (RMSE) and mean absolute error (MAE):

$$R^{2}=\frac{\sum_{i=1}^{N}{\left(Y\right)}^{2}+\sum_{i=1}^{N}{\left(Y-Y_{p}\right)}^{2}}{\sum_{i=1}^{N}{\left(Y\right)}^{2}}$$ (13)

$$RMSE=\sqrt{\frac{1}{N}\sum_{i=1}^{N}{\left(Y-Y_{p}\right)}^{2}}$$ (14)

$$MAE=\frac{1}{N}\sum_{i=1}^{N}\left|Y-Y_{p}\right|$$ (15)

where N is the number of data points, Y is the vector of outputs (*σ*_*x*_) in (12), and Y_p_ is the vector of predicted values. The higher R^2^ or the lower RMSE and MAE show that the performance is better.

**Fig 9:**
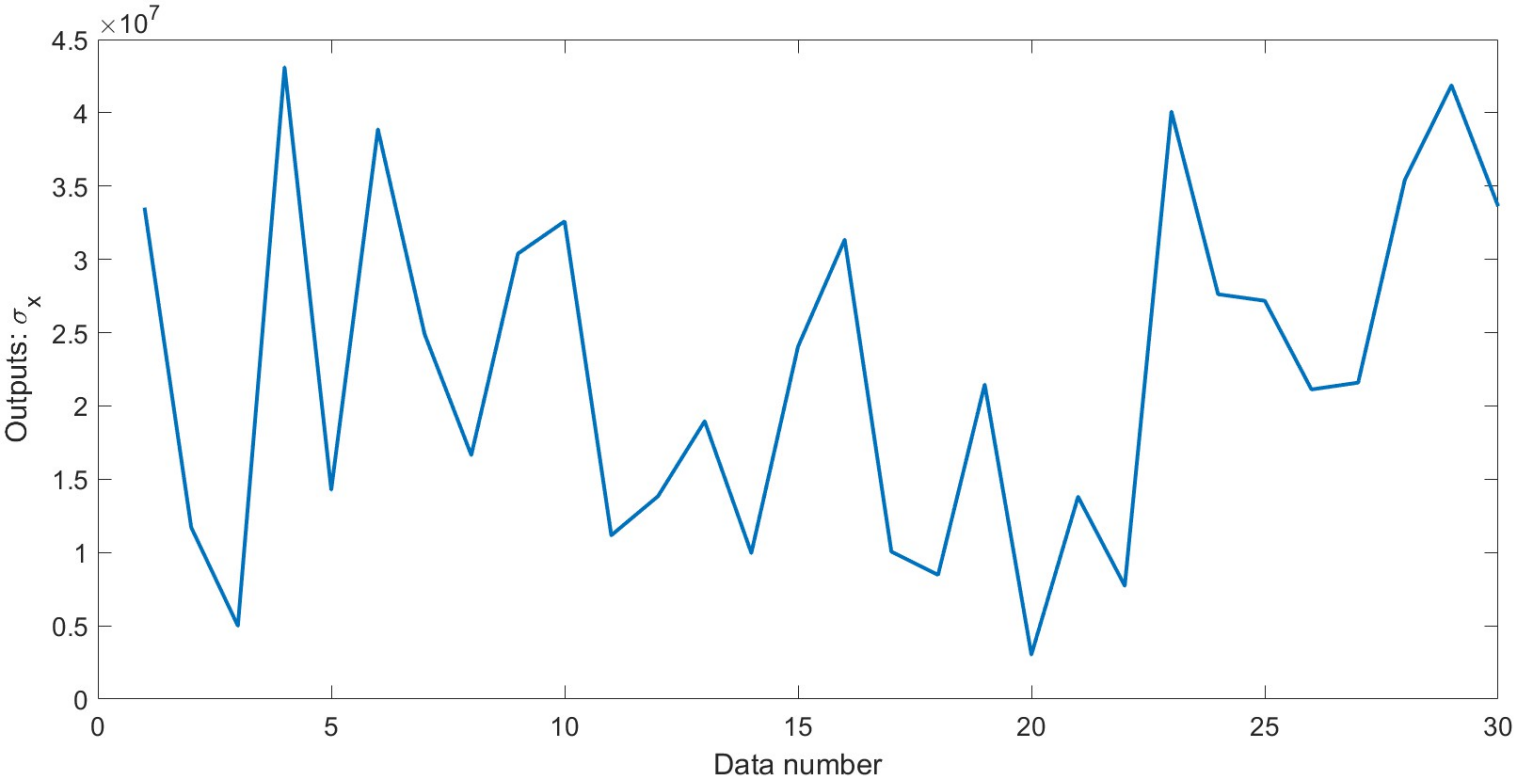
Outputs *σ*_*x*_ with errors in dataset 3.

**Fig 10:**
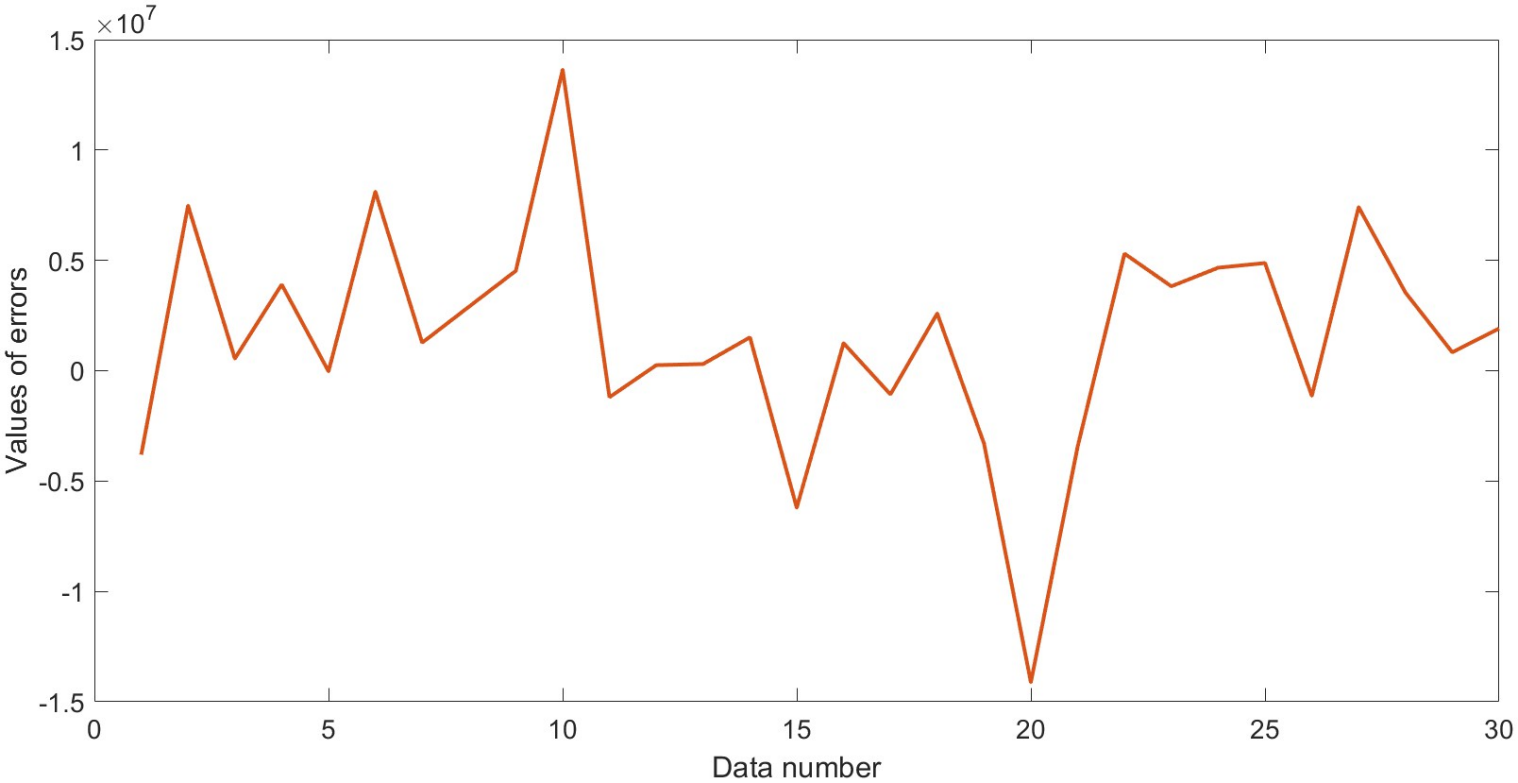
Values of errors in dataset 3.

**(a)** A 30-dataset (dataset 3: used for simulating the less data situation): 20 for the regression test and 10 for the prediction test were discussed. The statistics of dataset 3 are shown in Table 4, Figs 9 and 10.

**Table 4:** Statistics of the parameters and variables in dataset 3.

	*σ*_*x*_	*E*	*ε*_*x*_	*ε*_*y*_	*μ*
	(*10^7^ N/m^2^)	(*10^8^ N/m^2^)			
Maximum	4.310	3.959	0.097	0.0097	0.388
Minimum	0.304	2.127	0.007	0.00035	0.202
Mean	2.245	3.189	0.058	0.0048	0.301
Variance	1.384*10^7^	2.740*10^7^	0.00082	0.000008	0.0028

**Fig 11:**
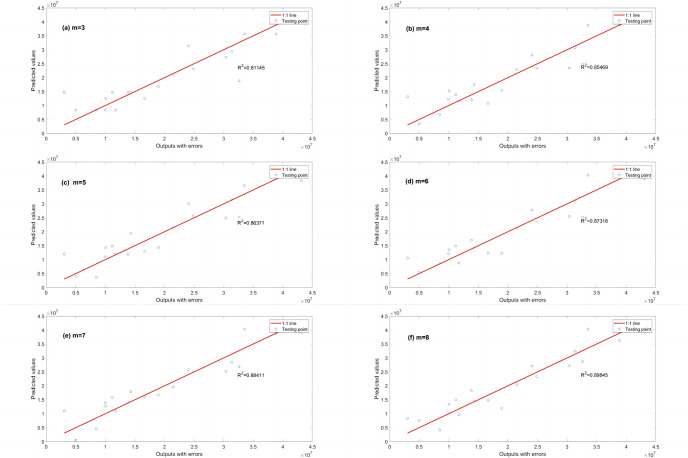
R^2^ calculated by EPR with PCS in dataset 3. (a) m = 3, (b) m = 4, (c) m = 5, (d) m = 6, (e) m = 7 and (f) m = 8.

**Fig 12:**
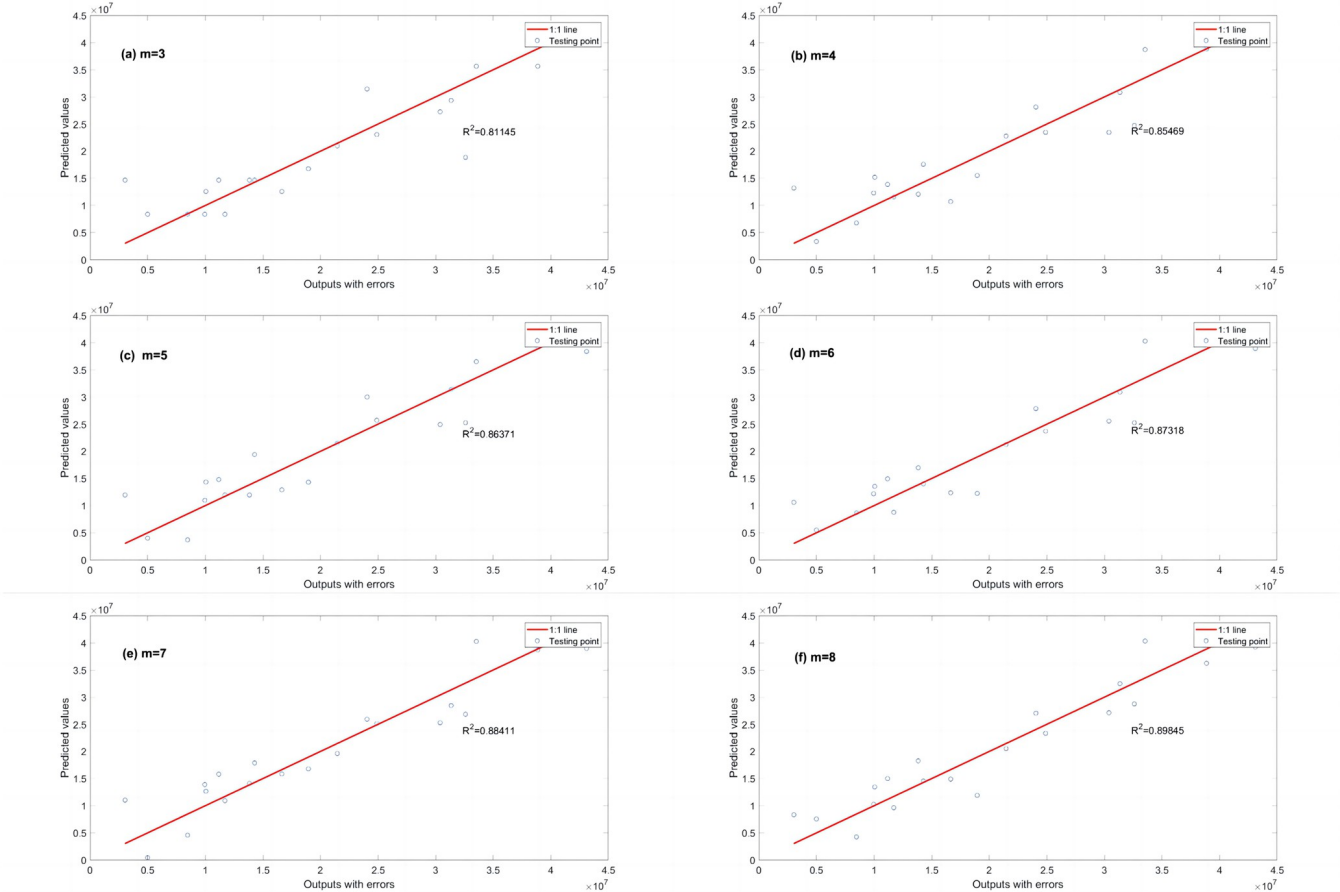
R^2^ calculated by EPR with L1RM in dataset 3. (a) m = 3, (b) m = 4, (c) m = 5, (d) m = 6, (e) m = 7 and (f) m = 8.

**Fig 13:**
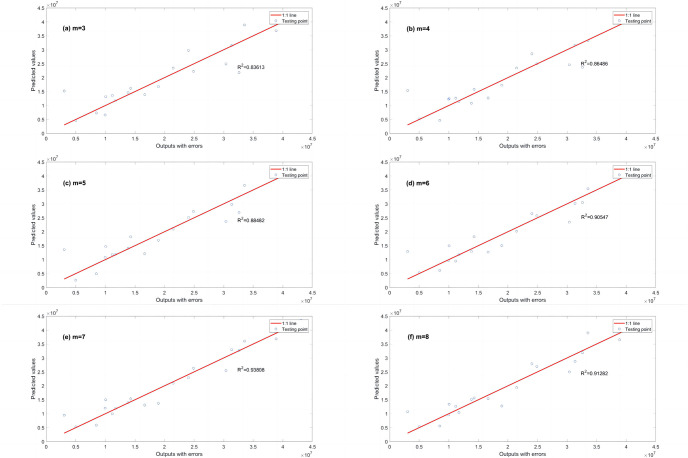
R^2^ calculated by EPR with L2RM in dataset 3. (a) m = 3, (b) m = 4, (c) m = 5, (d) m = 6, (e) m = 7 and (f) m = 8.

R^2^ is used to evaluate the regression performance of the three EPR models with different fitness functions: a classical fitness function (PCS) and two improved fitness functions L1RM and L2RM shown in Figs 11–13. R^2^ (calculated by EPR with the classical fitness function (PCS)) increases from 0.81145 to 0.89845 as the number of terms increases from 3 to 8 (Fig 11). Additionally, R^2^ (calculated by EPR and the improved fitness functions with L1RM) increases from 0.85774 to 0.95967 as the number of terms increases from 3 to 8 (Fig 12). Moreover, R^2^ (calculated by EPR with the improved fitness functions with L2RM) increases from 0.83613 to 0.93808 as the number of terms increases from 3 to 8 (Fig 13). The higher R^2^ demonstrates a better performance, revealing that the regression performance of EPR with L1RM is the best, and that of EPR with L2RM is better than the classical EPR, whose fitness function is PCS. After the regression performance is compared, the prediction performance (generalization ability) is discussed (Table 5) when the number of terms is 6 (m = 6).

**Table 5:** Prediction performance of 3 EPR models in dataset 3.

	EPR with PCS	EPR with L1RM	EPR with L2RM
EPR models	$\begin{array}{l} \sigma_{x}=-5.1\cdot 10^{7}+0.88E\mu \\ -6.5\cdot 10^{8}\varepsilon_{y}+1.78\cdot {\text{10}}^{\text{-5}}\frac{E\mu }{\varepsilon_{x}{}^{2}}\\ +1.96\cdot {\text{10}}^{\text{-7}}\frac{1}{\mu }+1.47E\mu -0.33E \end{array}$	$\begin{array}{l} \sigma_{x}=6.92\cdot 10^{6}-3.23\cdot 10^{-8}E^{2}\mu \varepsilon_{x}\\ -0.0001E^{2}\varepsilon_{x}\varepsilon_{y}{}^{2}+1.14\cdot {\text{10}}^{\text{-7}}E^{2}\mu^{2}\varepsilon_{x}\\ +1.88\cdot {\text{10}}^{\text{-9}}\frac{E^{2}\varepsilon_{y}}{\varepsilon_{x}}+3.31\cdot 10^{-21}{\left(\frac{E}{\varepsilon_{x}\varepsilon_{y}}\right)}^{2}\\ +1.18\cdot {\text{10}}^{3}\frac{E\varepsilon_{x}{}^{2}\varepsilon_{y}}{\mu } \end{array}$	$\begin{array}{l} \sigma_{x}=9.36\cdot 10^{6}+2.51\cdot 10^{-7}E^{2}\varepsilon_{x}\\ +1.51\cdot 10^{-6}E^{2}\mu^{2}\varepsilon_{x}-9.31\cdot {\text{10}}^{\text{-7}}E^{2}\mu \varepsilon_{x}\\ -\text{2}.17\cdot {\text{10}}^{\text{-8}}\frac{E^{2}\varepsilon_{x}}{\mu }-3.86\cdot 10^{-10}E^{2}\mu \\ +\text{2}.22\cdot {\text{10}}^{\text{-15}}\frac{E^{2}\mu }{\varepsilon_{x}\varepsilon_{y}} \end{array}$
R^2^	0.24437	0.34889	0.46732
RMSE	9186821.38	8527800.09	7713323.60
MAE	6116553.31	7646176.93	5647908.66

Table 5 reveals the prediction performances (generalization ability) of the three EPR models based on three indicators. EPR with L2RM ranks first and offers the best performance. EPR with L1RM ranks second, and EPR with PCS is third. The complexities of the three EPR models are different; the EPR model with PCS has lower variables (the value of *E*, *μ*, *ε*_*x*_, *ε*_*y*_ is 10), and EPR models with L1RM or with L2RM have more variables (the value of *E*, *μ*, *ε*_*x*_, *ε*_*y*_ for EPR models with L1RM and L2RM is 19 and 17, respectively). The EPR model with L1RM or with L2RM has smaller values of coefficients than the EPR model with PCS. The performance of 3 EPR models is different because PCS punishes the number of terms, and L1RM or L2RM punishes the values of coefficients. Thus, the data variability is high because the amount of data is small. Meanwhile, the R^2^ for regression is very different from R^2^ for prediction.

**Fig 14:**
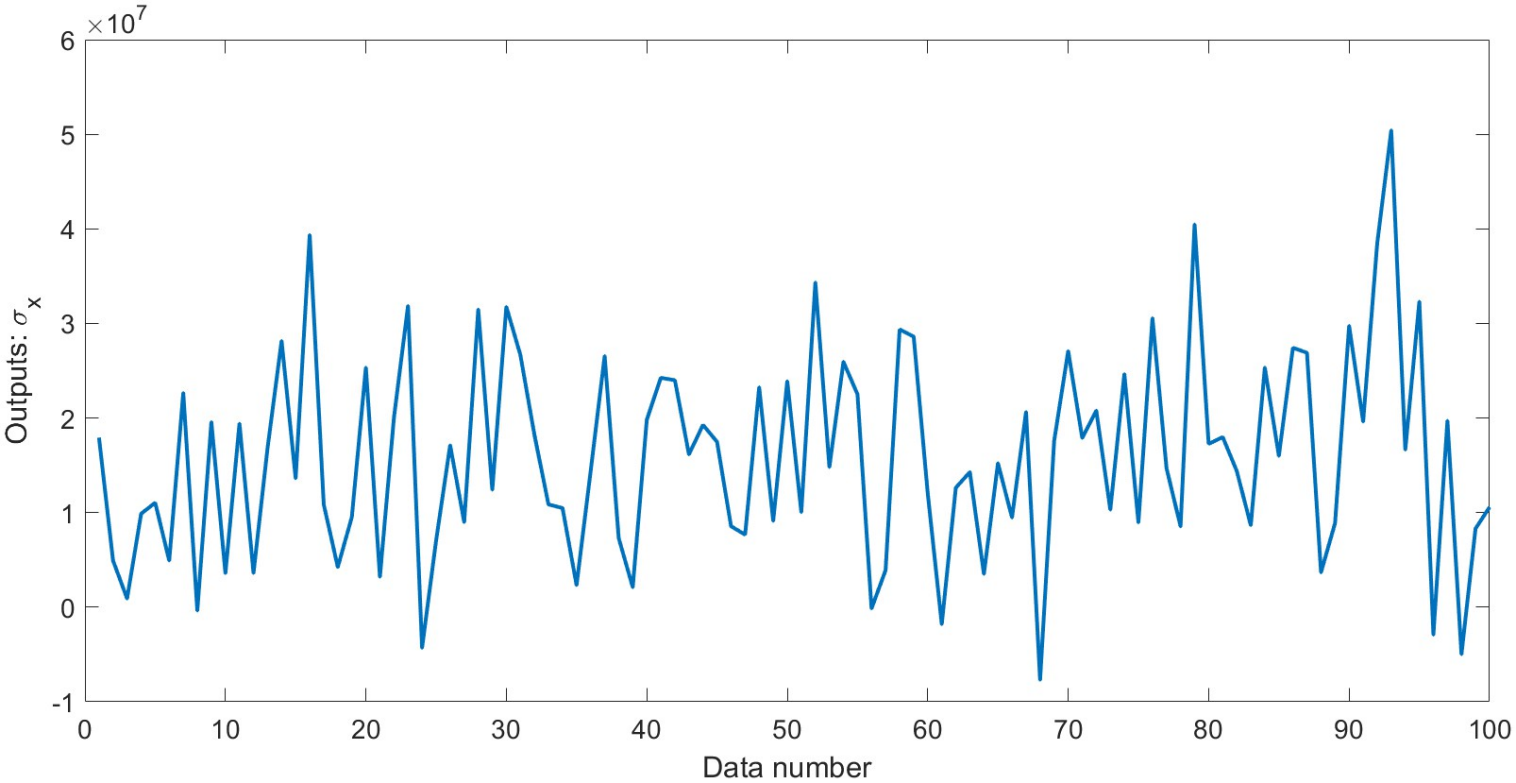
Outputs *σ*_*x*_ with errors in dataset 4.

**Fig 15:**
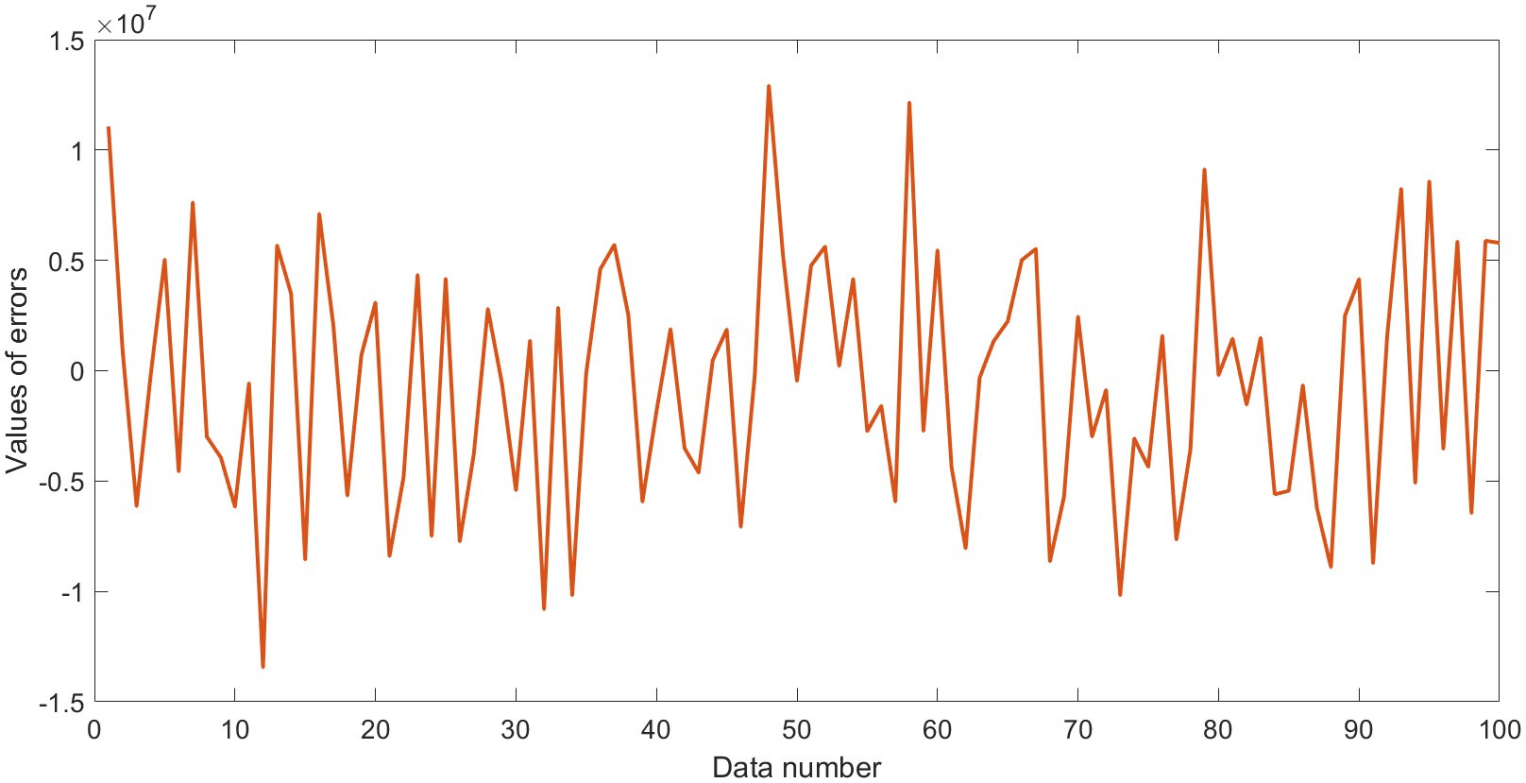
Values of errors in dataset 4.

**(b)** We discussed a 100-dataset (dataset 4: used for simulating the normal amount of data): 70 for the regression test and 30 for the prediction test. The statistics of dataset 4 are shown in Table 6, Figs 14 and 15.

**Table 6:** Statistics of the parameters and variables in dataset 4.

	*σ*_*x*_	*E*	*ε*_*x*_	*ε*_*y*_	*μ*
	(*10^7^ N/m^2^)	(*10^8^ N/m^2^)			
Maximum	5.042	3.985	0.097	0.001	0.398
Minimum	-0.770	2.035	0.002	0.00003	0.206
Mean	1.591	2.966	0.049	0.0054	0.302
Variance	1.219*10^7^	2.760*10^7^	0.00083	0.000008	0.0033

**Fig 16:**
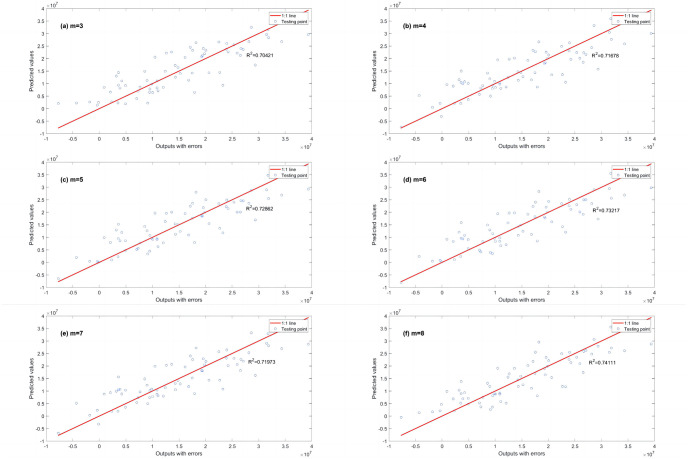
R^2^ calculated by EPR with PCS in dataset 4. (a) m = 3, (b) m = 4, (c) m = 5, (d) m = 6, (e) m = 7 and (f) m = 8.

**Fig 17:**
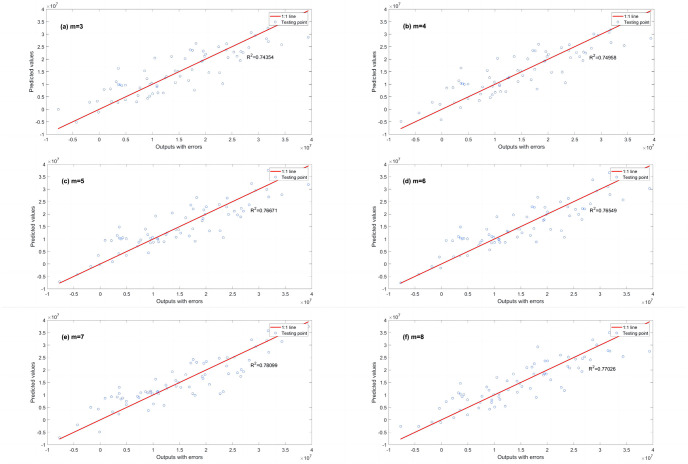
R^2^ calculated by EPR with L1RM in dataset 4. (a) m = 3, (b) m = 4, (c) m = 5, (d) m = 6, (e) m = 7 and (f) m = 8.

**Fig 18:**
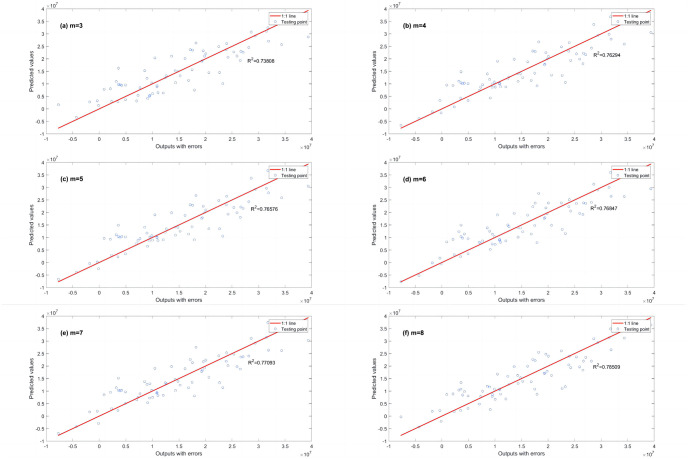
R^2^ calculated by EPR with L2RM in dataset 4. (a) m = 3, (b) m = 4, (c) m = 5, (d) m = 6, (e) m = 7 and (f) m = 8.

R^2^ was used to evaluate the regression performance of the three EPR models (with different fitness functions). The classical fitness function (PCS) and the two improved fitness functions with L1RM and L2RM) are shown in Figs 16–18. As the number of terms increases from 3 to 8, R^2^ (calculated by EPR with the classical fitness function (PCS)) increases from 0.70421 to 0.74111(Fig 16). R^2^ (calculated by EPR with the improved fitness functions with L1RM) increases from 0.74354 to 0.78099 as the number of terms increases from 3 to 8 (Fig 17). R^2^ (calculated by EPR with improved fitness functions with L2RM) increases from 0.73808 to 0.78509, as the number of terms increases from 3 to 8 (Fig 18). Thus, the regression performance of the EPR with L1RM and that of EPR with L2RM is close. Both models perform better than the classical EPR (whose fitness function is PCS). After comparing the regression performance, the prediction performance (generalization ability) is discussed (Table 7) when the number of terms is 6 (m = 6).

**Table 7:** Prediction performance of 3 EPR models in dataset 4.

	EPR with PCS	EPR with L1RM	EPR with L2RM
EPR models	$\begin{array}{l} \sigma_{x}=-7.0\cdot 10^{7}+1.11\cdot {\text{10}}^{7}\frac{\text{1}}{\mu }\\ +1.43\cdot 10^{8}\mu -2.45\cdot {\text{10}}^{4}\frac{\text{1}}{\varepsilon_{x}}\\ -1.2\cdot {\text{10}}^{\text{23}}\frac{1}{E^{2}}+0.93E\varepsilon_{x} \end{array}$	$\begin{array}{l} \sigma_{x}=8.4\cdot 10^{6}-0.0011\frac{\text{1}}{\mu \varepsilon_{x}\varepsilon_{y}{}^{2}}\\ +2.99\cdot 10^{-8}E^{2}\varepsilon_{x}{}^{2}+0.025\frac{\mu^{2}}{\varepsilon_{x}\varepsilon_{y}{}^{2}}\\ -1.22\cdot {\text{10}}^{\text{-16}}\frac{E^{2}}{\mu \varepsilon_{x}\varepsilon_{y}}+4.95\cdot 10^{-14}\frac{E^{2}\mu }{\varepsilon_{y}}\\ -6.6\cdot {\text{10}}^{4}\frac{\mu \varepsilon_{y}}{\varepsilon_{x}{}^{2}} \end{array}$	$\begin{array}{l} \sigma_{x}=5.7\cdot 10^{6}+1.41\cdot 10^{-9}{\left(\frac{E\varepsilon_{x}}{\mu }\right)}^{2}\\ +6.99\cdot 10^{\text{-17}}\frac{E^{2}}{\mu^{2}\varepsilon_{y}}-6.74\cdot {\text{10}}^{\text{-22}}\frac{E^{2}}{\mu \varepsilon_{x}{}^{2}\varepsilon_{y}{}^{2}}\\ +4.61E\mu^{2}\varepsilon_{x}+0.0021{\left(\frac{\mu }{\varepsilon_{x}\varepsilon_{y}}\right)}^{2}\\ -1.1\cdot {\text{10}}^{\text{-6}}\frac{E\mu }{\varepsilon_{x}} \end{array}$
R^2^	0.76728	0.81959	0.8087
RMSE	5901792.48	5196360.01	5350863.83
MAE	4880189.75	4329662.19	4456582.20

As shown in Table 7, the prediction performances (generalization ability) of the three EPR models presented three indicators. EPR with L1RM offers the best performance; EPR with L2RM ranks second. However, its performance is close to EPR with L1RM. EPR with PCS is the third. R^2^ calculated by EPR with L1RM is 6.4%, which is higher than that of the EPR with PCS. Moreover, the RMSE of EPR with L1RM is 12.0% lower than that of EPR with PCS, and the MAE of EPR with L1RM is 11.3%, which is lower than that of EPR with PCS. The complexities of the three EPR models are still different. The EPR model with PCS has fewer variables (the value of *E*, *μ*, *ε*_*x*_, *ε*_*y*_ is 6); the EPR models with L1RM or L2RM have more variables (the value of *E*, *μ*, *ε*_*x*_, *ε*_*y*_ is 18 and 19 for L1RM and L2RM, respectively). Note that the EPR model obtained by EPR with PCS does not have the term of *ε*_*y*_. Although the results are relatively accurate, they miss the data of variables *ε*_*y*_; such a model is vulnerable. R^2^ for regression is close to R^2^ for prediction because the amount of data is normal.

## 4. Conclusions

In this study, EPR significantly improved using L1RM and L2RM regularization methods. We built testing datasets that were used for simulating geotechnical engineering data. Appropriate values of generalization parameters (λ) in L1RM and L2RM were first studied and determined. Then, the regression ability and generalization ability of EPR with both regularization methods were tested and compared with that of EPR with PCS in various conditions, i.e., the less data situation and normal data situation.

By punishing the number of terms, EPR with PCS (the classic EPR) controls the complexity of its model. Moreover, the EPR with L1RM or L2RM controls the complexity of its model by punishing the coefficient values of the model. Therefore, the models obtained by EPR with PCS had fewer terms and were more concise. However, they lost independent variables making them suffer from potential dangers. Models obtained by EPR with L1RM or L2RM had more terms and lower coefficient values. Although they seemed to be more complex, they contained all the independent variables. Furthermore, lower coefficient values could promote the generalization ability of EPR models and help avoid overfitting.

When dealing with the less data situation, EPR with L1RM or L2RM had better regression and prediction abilities (generalization ability) than EPR with PCS. When dealing with the normal data situation, EPR with L1RM had the best regression ability and prediction ability. EPR with L2RM was still better than EPR with PCS in both regression and prediction abilities. In summary, both EPR with L1RM and L2RM perform better than EPR with PCS in regression abilities and generalization ability, and EPR with L1RM performs a little better than EPR with L2RM.

Thus, both the regression ability and generalization ability of EPR are promoted by introducing regularization methods (L1RM and L2RM). Moreover, the vulnerability of losing some independent variables of the classic EPR is eliminated, so the EPR is improved through regularization methods.
